# Patterns and Structures of Land Use Change in the Three Rivers Headwaters Region of China

**DOI:** 10.1371/journal.pone.0119121

**Published:** 2015-03-27

**Authors:** Jingbiao Yang, Yi Chen Wang, Luo Guo, Dayuan Xue

**Affiliations:** 1 School of Nature Conservation, Beijing Forestry University, Beijing, China; 2 Department of Geography, National University of Singapore, Singapore, Singapore; 3 College of Life and Environmental Sciences, Minzu University of China, Beijing, China; Yale School of Public Health, UNITED STATES

## Abstract

Located in Qinghai Province of China, the Three Rivers Headwaters Region is the source region of the Yangtze, Yellow and Lantsang Rivers, and plays an important role in biodiversity conservation and regulating water supply. Despite many efforts on land use change in Qinghai, knowledge of the spatial variation of land use change is still lacking. This study examines the patterns of land use change across various watersheds, prefectures and the temple surroundings. Remote sensing images of 1987, 1997 and 2007 were analyzed to derive land use distributions; patterns and structures of the landscape were then quantified with landscape metrics. The results illustrated that the Yangtze River headwater region had more diverse and more evenly distributed landscape, while the Lantsang and the Yellow headwater regions showed a decline in landscape diversity. Comparison of the land use patterns of four prefectures revealed that Yushu Prefecture experienced an increase in landscape diversity from 1987 to 2007 while the land use patches in Guoluo Prefecture exhibited more aggregated patterns than other prefectures. Analysis of the spatial variations of land use change in the temple surroundings illustrated that 19.7% and 35.9% of the temples in Guoluo and Yushu Prefectures, respectively, encountered land use change for their immediate areas within 2 km. Comparison of the surroundings of temples and human settlements found that land use change was not evenly distributed, and that greater land use change had occurred for the surroundings of human settlements. Such findings provided insights into the spatial variation of land use change in the Three Rivers Headwaters Region.

## Introduction

Rapid population growth and continuous exploitation of natural resources during the past century have altered the Earth system. Through land use, humans have modified over 83% of the terrestrial surface [1,2]. Indeed, land use change is one of the most essential interacting components of global change affecting the Earth system [3,4]. Numerous research has revealed that land use change has major effects on climate, hydrologic regimes, ecosystems, and human welfare [2,5–7]. In particular, conversion of land from more natural conditions to less natural conditions in headwater regions is of concern. Not only because headwater regions contain critical habitats for biota [8], but also that land use change in these regions can have cascading impacts on downstream ecosystem functioning [9,10].

In China, the Three Rivers Headwaters Region (TRHR) is the source region for the Yellow, the Yangtze, and the transnational Lantsang-Mekong Rivers, thereby playing an important role in regulating regional water supply and climate of East Asia. Located in the Qinghai-Tibet Plateau with an average altitude of 4000 m, the TRHR houses many endemic species while its alpine ecosystems are very vulnerable to disturbances [11]. Although the TRHR is now a nature reserve for biodiversity conservation, human-induced land use change has persisted in the last several decades [12]. Considerable work has therefore been done with regard to assessing land use change in the TRHR. Studies have analyzed remote sensing images and various geospatial data, such as topographic maps and field surveys, to quantify land use and vegetation cover change [12–16], investigate grassland degradation [17–20], and examine spatial pattern and structure of ecosystem dynamics [21]. Recent work has incorporated landscape ecology concepts to explore the patterns and structures of land change [22–24].

Despite these research efforts, patterns of land use change in the TRHR are still not adequately addressed. Prior studies have focused on the headwater region for one of the three rivers. Comparative analyses for all the three rivers or across different regions in the TRHR are needed to understand the spatial variations of change. In addition, little information is available on the influences of temples on land use change. As the TRHR is located in the Qinghai-Tibet Plateau where the villagers have strong cultural believe in Tibetan Buddhism and temples have been the centers of ceremonies and activities for many centuries, there is a need to scrutinize the patterns of land use change in the surrounding areas of the temples.

The aim of this study is to examine patterns of land use change in the TRHR with two specific questions. First, do land use patterns exhibit different structures of change across various watersheds and autonomous prefectures in the TRHR? Second, do the surrounding areas of the temples in different regions of the TRHR show different patterns of land use change? By comparing the patterns and structures of landscape change in the TRHR and considering the influences of temples and local cultural belief, the study contributes to the understanding of the spatial variations of land change in the TRHR.

## Materials and Methods

### Study Area

**Fig 1:**
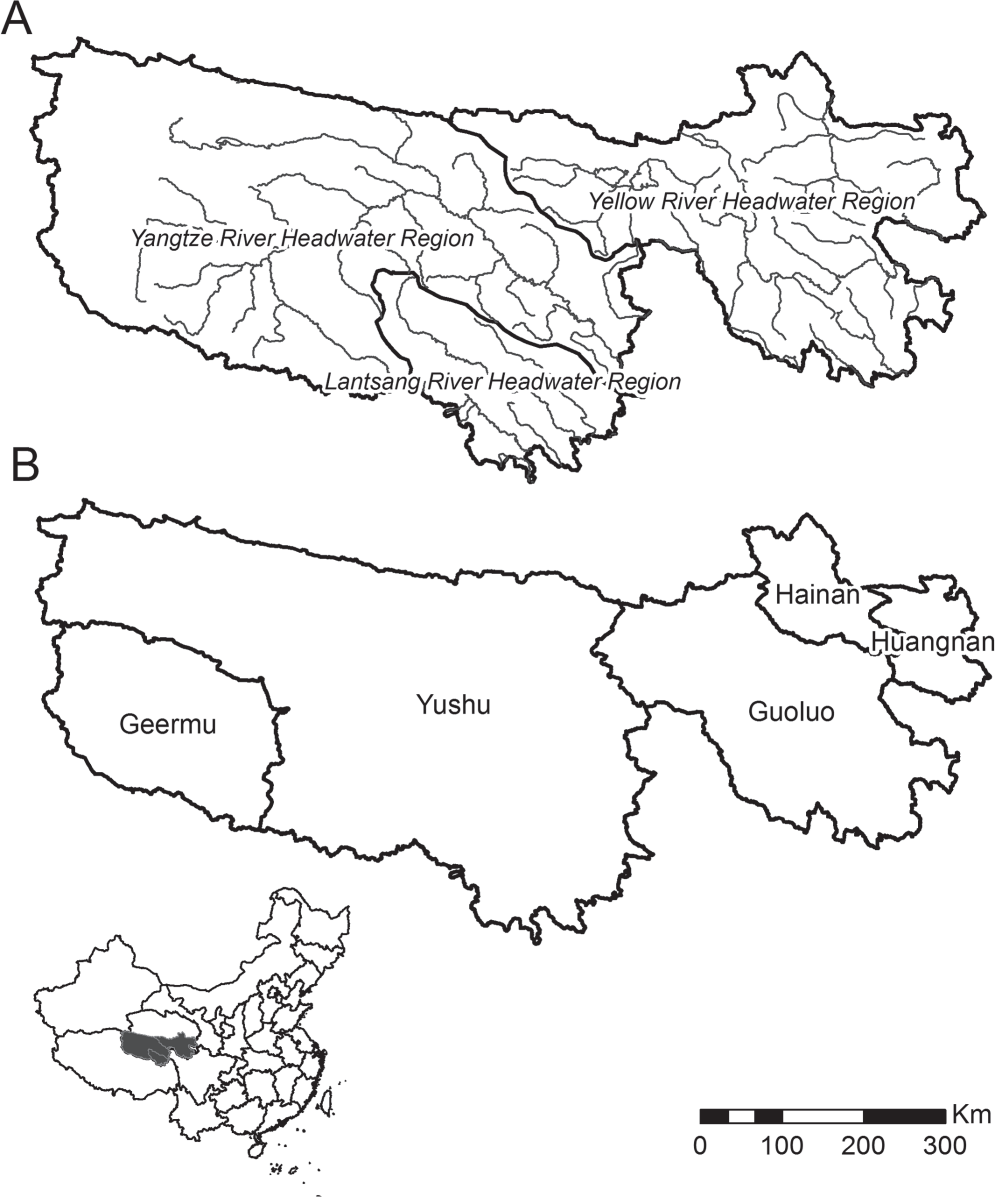
The study area of the Three River Headwaters Region. (A) Distributions of the Yangtze River, Lantsang River, and Yellow River headwater regions. (B) Locations of Hainan Prefecture, Huangnan Prefecture, Guoluo Prefecture, Yushu Prefecture, and Geermu City.

The TRHR, also known as “Sanjiangyuan”, is located in southern Qinghai Province, western China(Fig. 1).The study area lies between 31°39′N and 36°16′Nand 89°24′E and 102°23′E, covering an area of approximately 3.58×10^5^ km^2^, with elevation ranging from 3450 m to 6621 m. The TRHR has been described as “the Water Tower of China and Asia”[25] because several large rivers all originate in this region. The area is characterized by a cold and dry climate. Annual mean temperature ranges between -5.6°Cand 3.8°C, and annual mean precipitation between 262.2 mm and 772.8 mm [11].

The study area includes all or some of the counties from four Tibetan Autonomous Prefectures, which are Yushu Prefecture, Guoluo Prefecture, Hainan Prefecture, and Huangnan Prefecture, and one township, which is Tanggula Township of Geermu City (Fig. 1). The Tibetan ethnic group consists of about 90% of the population. The religion of most of the population is Buddhism, which was introduced to the region about 1400 years ago [26]. Over the past hundreds of years, many temples have been constructed as the religious centers for ceremonies and activities. Temples are perceived as the Holy Lands of the Tibetans, and by protecting the sacrosanct temples and their surrounding environments, Tibetans express their worship of religion.

### Data and Land Use Classification

To examine patterns of land use change in the TRHR, Landsat Thematic Mapper (TM) and Enhanced-Thematic Mapper (ETM) images in the years of 1987, 1997, and 2007 were obtained from the Land Processes Distributed Active Archive Center (LPDAAC) of the U.S. Geological Survey for supervised land use classification. These three years of images were chosen for two reasons. First, most of them were free of cloud coverage. Second, the Qinghai provincial government officially announced a series of environmental protection actions in December 1996, including the conservation of the natural resources in the TRHR [27]. The time period between 1987 and 2007 was about one decade before and after the announcement and might provide insights into the patterns of change.

The Landsat TM/ETM images, after supervised classification, were then analyzed in conjunction with a 1:250 000 scale topographic map and thematic maps of soil and vegetation of the study area, with an object-based image classification method. Compared to traditional pixel-based classification methods, the object-based classification method incorporated multi-scale image segmentation and additional attributes, such as shapes and textures, and hence was more suited to landscape scale analysis [28]. Considering the classification schemes used previously in different areas of the TRHR [12–16,22–24,26], two levels of classification were derived (Table 1). The primary class consisted of six land use types, including farmland, forest, grassland, water, built-up land, and unused land. Each primary class was further divided into several secondary classes, resulting in a total of 25 secondary classes. Classification was done in ERDAS IMAGINE 8.7 [29].

**Table 1:** Land use classification system for the Three Rivers Headwaters Region.

Primary class	Secondary class
Farmland	Paddy field
	Dry land
Forest	Woodland
	Shrub
	Open forest
	Other forest
Grassland	High coverage grassland
	Middle coverage grassland
	Low coverage grassland
Water	River
	Lake
	Reservoir
	Glacier and firn
	Mudflat
	Bottomland
Built-up land	Town land
	Rural settlement
	Other
Unused land	Sand
	Gobi
	Salt lick
	Frozen fenland
	Bareland
	Exposed rock and shingle land
	Other

Each primary class is further classified into several secondary class.

### Quantifying Landscape Structures with Landscape Metrics

To quantify the landscape structures of change in different watersheds and autonomous prefectures across the three time periods, the classification results from remote sensing analysis were then analyzed and quantified using landscape metrics in FRAGSTATS 3.3 [30].A total of four landscape metrics were calculated, including Interspersion and Juxtaposition Index (IJI), Contagion Index (CONTAG), Shannon’s Diversity Index (SHDI), and Shannon’s Evenness Index (SHEI).These metrics provided quantitative measurements of the configuration and diversity of the land use patches of the landscape [23,30,31]. Analyses were carried out for the TRHR study area as a whole, followed by comparisons of different watersheds (i.e., Yangtze, Lantsang, and Yellow Rivers) and different autonomous prefectures (i.e., Yushu, Guoluo, Hainan, and Huangnan Prefectures).

### Examining Land Use Change in Temple Surroundings

To examine the patterns of land use change in the surrounding areas of temples, location data of temples were acquired and then digitized into ArcGIS 9.2 [32].With point locations of temples as the centers, buffer zones of 2km, 4km and 6km were constructed using the Spatial Analyst Extension in ArcGIS to derive three buffer zones radiated from the centers. The radius of 2km was within most villagers’ walking distance, particularly for agricultural activities, while the radius of 6km was approximately the maximum distance that villages could manage their free range herds. The three buffer zones (i.e., 0–2 km, 0–4 km, and 0–6 km) were then overlaid with the land use distributions of 1987, 1997 and 2007 to calculate the percentages of temples that experienced land use change.

Analyses were done for Yushu and Guoluo Prefectures because they have longer histories of Tibetan Buddhism influences. In addition, location data of temples were acquired for these two prefectures as a result of data availability. Nevertheless, the two prefectures consisted of about 78% of the study area, and hence provided a representative data set for the TRHR.

## Results and Discussion

### Changes of landscape structures

#### TRHR

The patch adjacency of various land use patches in the TRHR, indicated by IJI, decreased from 1987 to 1997 and then increased from 1997 to 2007, but overall, the change exhibited a declining trend (Table 2). Conversely, the extent to which land use patch types were aggregated, indicated by CONTAG, increased from 1987 to 1997, followed by a decrease afterward, but overall, the change showed a slight increasing trend (Table 2). The overall decline of IJI and increase of CONTAG suggested that the land use patches in the TRHR became less equally adjacent to each otherandmore spatially aggregated. An overall declining trend was observed for both the SHDI and SHEI, suggesting a decrease in landscape heterogeneity (Table 2). Indeed, during the period of the study, the number and areas of farmland increased, taking up much of the water resources that had already been limited to the region. Subsequently, desertification and grassland degradation occurred, resulting in an increase in bare land and gobi.

**Table 2:** Landscape structure change of the Three Rivers Headwaters Region.

Year	IJI	CONTAG	SHDI	SHEI
1987	42.2748	54.0800	0.8926	0.4982
1997	42.0910	54.6056	0.8802	0.4913
2007	42.2138	54.3207	0.8845	0.4936

IJI: Inspersion and Juxtapositon Index; CONTAG: Contagion Index; SHDI: Shannon's Diversity Index (SHDI); SHEI: Shannon's Evenness Index.

#### By Three Watersheds

Land use distributions across the headwater regions of the Yangtze, Lantsang, and Yellow Rivers exhibited different patterns of change (Table 3). For the Yangtze and the Lantsang Rivers, IJI respectively increased and decreased between 1987 and 2007. For the Yellow River, IJI decreased for the first decade of analysis from 1987 to 1997, and then increased for the second decade of analysis, but overall the value declined. The trends of change for CONTAG showed an increase for the Lantsang River from 1987 to 2007, but for the Yangtze and the Yellow Rivers, the values increased first from 1987 to 1997 and then declined from 1997 to 2007. Different from the Yangtze and the Yellow Rivers, the SHDI and SHEI for the Lantsang River decreased during the period of study.

**Table 3:** Comparison of landscape structure change of the Three Rivers Headwaters Region by watersheds.

Watershed	Year	IJI	CONTAG	SHDI	SHEI
Yangtze River	1987	54.7412	46.5743	1.8268	0.591
	1997	55.8822	47.3119	1.8195	0.5886
	2007	57.3194	45.9238	1.8305	0.6012
Lantsang River	1987	42.9180	54.8161	1.4344	0.4788
	1997	42.6800	56.0318	1.3973	0.4664
	2007	42.6663	56.2508	1.3891	0.4637
Yellow River	1987	52.0027	45.9313	1.8033	0.5834
	1997	49.7610	47.4961	1.7489	0.5658
	2007	50.8792	46.4599	1.7567	0.5770

The declines in IJI, SHDI, and SHEI for the Lantsang River signified a transformation to a less heterogeneous landscape. It was observed that spatial distribution of land use patches in the Lantsang River headwater region changed from a scattered pattern into aggregated patches, and that the land use patches of low coverage grassland and medium coverage grassland were better connected. These findings were in agreement with the increasing values of CONTAG, because higher values of CONTAG might result from landscape with a few large, contiguous patches [30]. On the contrary, the SHDI and SHEI for the Yangtze and the Yellow Rivers both decreased first, followed by an increase, although the SHDI for the Yellow River in 2007 was still lower than that in 1987. This indicated that a decrease in diversity with uneven distribution of the land use patches for the two headwater regions from 1987 to 1997, and then an increase in the diversity of patches with improved evenness from 1997 to 2007. The decline of CONTAG between 1997 and 2007 for these two headwater regions further supported that the land use patches were more interspersed after 1997.

#### By Four Prefectures

Analyses of the landscape structures for the four Tibetan Autonomous Prefectures showed that Hainan Prefecture and Huangnan Prefecture had similar patterns of change. Both prefectures, located in the eastern part of the study area, experienced continuous decreasing trends for IJI, SHDI, and SHEI (Table 4), illustrating that the structures of their land use patches became less equally adjacent and less evenly distributed. The change was probably caused by the growing dominance of low coverage grassland and medium coverage grassland. Alternatively, although the IJI for Yushu Prefecture decreased from 1997 to 2007, the overall change for the period of study showed arising trend as the value in 2007 was greater than that in 1987. This denoted that all the land use patch types in Yushu Prefecture were more equally adjacent to each other than those in the other three prefectures. Yushu Prefecture was also the only prefecture with an increase in SHDI from 1987 to 2007, indicating that its landscape had become more diverse than the other three prefectures. The increase in CONTAG for Yushu Prefecture, however, revealed that the patches of some of its land use classes might have become more aggregated. In contrast, although the CONTAG values for Guoluo Prefecture for the three time periods were greater than most of the other prefectures, the decline in CONTAG from 1997 to 2007 for Guoluo illustrated a reverse change to more fragmented landscape because a lower CONTAG generally characterized the landscape with many small and dispersed patches.

**Table 4:** Comparison of landscape structure change of the Three Rivers Headwater Regions by prefectures.

Prefecture	Year	IJI	CONTAG	SHDI	SHEI
Yushu	1987	56.7021	43.1304	1.8917	0.6214
	1997	57.6430	43.1464	1.9043	0.6255
	2007	57.3317	43.3905	1.9183	0.6206
Guoluo	1987	51.4501	46.9072	1.7611	0.5785
	1997	47.3162	50.0447	1.6496	0.5418
	2007	48.4082	48.8743	1.6600	0.5541
Hainan	1987	57.5847	43.2800	1.8808	0.6388
	1997	56.6464	43.5631	1.8681	0.6344
	2007	54.3426	45.6591	1.8674	0.6134
Huangnan	1987	56.4802	47.4497	1.8648	0.6125
	1997	55.7623	50.6543	1.7602	0.5782
	2007	55.6287	50.6440	1.7517	0.5754

### Surrounding Areas of Temples

#### Guoluo Prefecture

A total of 66 temples were located in Guoluo Prefecture. In 1987, most of the temples were surrounded by grassland related land use types, of which 20% were located in the high coverage grassland, 64% in the medium coverage grassland, and 16% in the low coverage grassland. Extractions of the land use patches showed that within the 2 km buffer zone, there were usually 2 to 5 different land use types, most of which were grassland. Within the 4 km buffer zone, in addition to grassland related land use types observed for the 2 km buffer zone, forest and unused land use types were noted, including forest land, open forest, shrub, bare land, and bare rock land. The pattern suggested a transition from grassland dominated landscape immediately surrounding the temples to forest or unused land further away from the temples. Within the 6 km buffer zone, apart from the land use types found within the 4 km radius of the temples, some other land use types were spotted.

Analyses of land use change revealed that between 1987 and 2007, 13 (19.7%), 8 (12.1%), and 6 (9.0%) of the temples in Guoluo Prefecture encountered land use change for their surrounding areas within the 2 km, 4 km and 6 km buffer zones, respectively (Table 5). The result suggested that more temples experienced land use change in their immediate surrounding environments within 2 km, while the number of temples that experienced land use change decreased as the size of the buffer zone increased. The findings indicated the possibility of more human activities contributing to land use change in the areas that were closer to the temples than those that were further away.

**Table 5:** Number and percent of temples that experienced land use change.

Prefecture	2km	4km	6km
Guoluo	13 (19.7%)	8 (12.1%)	6 (9.0%)
Yushu	65 (35.9%)	31 (17.1%)	47 (26.0%)

The zones of 2 km, 4 km and 6 km indicate that land use change has occurred in these respective buffer zones of temples in Guoluo Prefecture and Yushu Prefecture. Percentages are calculated based on the total number of temples analyzed for Guoluo and Yushu, which are 66 and 181, respectively.

#### Yushu Prefecture

A total of 181 temples were located in Yushu Prefecture. Similar to Guoluo Prefecture, most of the temples in Yushu Prefecture were surrounded by grassland related land use types in 1987, but overall, 2 to 7 different land use types were observed for the buffer zone of 2 km for Yushu Prefecture. For the 4 km and 6 km buffer zones, in addition to the dominance of grassland related land use types, forest and unused land use types were found. Analyses of the patterns of land use change revealed that between 1987 and 2007, 65 (35.9%), 31 (17.1%), and 47 (26.0%) of the temples in Yushu Prefecture encountered land use change for their surrounding areas within 2 km, 4 km, and 6 km, respectively (Table 5). The percent of temples experiencing land use change for Yushu Prefecture was higher than that for Guoluo Prefecture. Furthermore, different from the patterns observed for Guoluo Prefecture, the number of temples that experienced land use change in Yushu Prefecture did not exhibit a continuous declining trend as the size of the buffer zone increased. This suggested the possibility of land use change further away from the temples in Yushu Prefecture.

#### Comparison with the Surroundings of Human Settlements

Land use change in the surroundings of temples were further compared with the surroundings of human settlements in Guoluo and Yushu Prefectures to understand if the temples or the settlements experienced different changes across the three time periods. Buffer zones were constructed around the human settlements to obtain their land use distributions in 1987, 1997 and 2007. The findings of the 4km buffer zones were used here as an indicative comparison for the surrounding areas of temples and settlements.

Among the 65 settlements analyzed for Guoluo Prefecture, 27 settlements (41.5%) had land use change detected between 1987 and 2007 (Table 6). This percent value was much higher than that of 12.1% for the 4km buffer zones of the temples in the same prefecture (Table 5). A close examination of the locations of the settlements revealed that most of the changes happened in the settlements located nearby main roads. A total of 24 and 28 settlements were distributed nearby main roads and temples, respectively (Table 6), but 18 (i.e., 75.0%) of the roadside settlements experienced land use change during the time period of analysis, as opposed to 8 (28.6%) of the settlements that were located nearby temples.

**Table 6:** Number of human settlements found with land use change in Guoluo and Yushu.

Prefecture	Settlements	Near roads	Near temples	Near lakes or rivers	Total
Guoluo	Analyzed	24	28	13	65
	Found change	18	8	1	27
Yushu	Analyzed	17	8	18	43
	Found change	10	5	7	22

The buffer zones of 4 km of the human settlements in Guoluo Prefecture and Yushu Prefecture are analyzed. Settlements are categorized based on whether their locations are close to main roads, temples, or water bodies of lakes or rivers.

Conversely, among the 43 settlements analyzed for Yushu Prefecture, 22 settlements (51.2%) experienced land use change between 1987 and 2007 (Table 6). This percent value was also much higher than that of 17.1% for the temples in Yushu Prefecture. Among the 43 settlements, 17 were located nearby main roads, 8 nearby temples, and 18 nearby lakes or rivers. Land use change was detected in 10 out of the 17 roadside settlements (58.8%), and 5 out of the 8 settlements located nearby the temples (62.5%). The higher proportions of both temples and settlements experienced more changes in their surroundings in Yushu Prefecture (Tables 5 and 6) might be due to the long established religious activities. The Tangbo Old Road, which extended from central China to Tibet, went through the prefecture, with many temples established along the road. The temples were not only the places for religious activities, but also for education. Also, as many temples in Yushu Prefecture are located in the south of the prefecture where population density is high, more human activities are expected to impact the surrounding environments of the temples.

## Conclusions and Future Work

This study analyzed the patterns and structures of land use change in the TRHR in 1987, 1997, and 2007. Overall, the structures of the land use across the three time periods remained similar, although a slight decrease in landscape diversity was noted. Comparisons of the land use change across various regions in the TRHR, however, showed different patterns. Comparison of the three headwater regions of the Yangtze, Lantsang and Yellow Rivers showed that the land use patches in the Yangtze River headwater region became more diverse and more evenly distributed between 1987 and 2007, while the land use patches in the headwater regions of the Lantsang and the Yellow Rivers were more unevenly distributed with a decline in landscape diversity. Comparison of the landscape patterns of Yushu, Guoluo, Hainan and Huangnan Tibetan Autonomous Prefectures revealed that the patterns of change were similar for Hainan and Huangnan Prefectures, both located in the eastern side of the TRHR. Conversely, among the four prefectures, only Yushu Prefecture experienced an increase in landscape diversity from 1987 to 2007 and the land use patches in Guoluo Prefecture exhibited more aggregated patterns than most of the other prefectures. Analysis of the spatial variations of land use change in the surrounding environments of the temples illustrated that 19.7% of the temples in Guoluo Prefecture encountered land use change for their immediate areas within 2 km. The number was much higher for Yushu Prefecture, of which 35.9% of its temples experienced land use change in their 2 km surroundings. Comparison of the surrounding areas of the temples and human settlements found that greater land use change had occurred during the time period of analysis for the surroundings of human settlements than those of the temples. For Guoluo Prefecture, 41.5% of the settlement surroundings of the 4 km buffer zone were detected with land use change, as opposed to 12.1% of the temples; for Yushu Prefecture, 51.2% of the settlement surroundings encountered change, as opposed to 17.1% of the temples.

The study provided insights into the spatial variation of land use change in the TRHR across different watersheds and prefectures. Future research can be conducted to further investigate the factors accounting for the differences in land use change surrounding the temples and the human settlements. In particularly, research can focus on how physical factors such as topography, how social-economic factors such as transportation development, and how cultural factors such as religious believe of Tibetan Buddhism in achieving the harmony between humans and the nature, interact to influence the patterns of land change in the TRHR.

## Supporting Information

S1 File Locations of the temples in Guoluo and Yushu Prefectures in GIS shapefile format. (RAR)
